# Gene therapy knockdown of VEGFR2 in retinal endothelial cells to treat retinopathy

**DOI:** 10.1007/s10456-018-9618-5

**Published:** 2018-05-05

**Authors:** Aaron B. Simmons, Colin A. Bretz, Haibo Wang, Eric Kunz, Kassem Hajj, Carson Kennedy, Zhihong Yang, Thipparat Suwanmanee, Tal Kafri, M. Elizabeth Hartnett

**Affiliations:** 1 John A. Moran Eye Center, University of Utah, 65 N. Mario Capecchi Drive, Salt Lake City, UT 84132 USA; 2 Gene Therapy Center, University of North Carolina at Chapel Hill, Chapel Hill, NC USA; 3 Department of Microbiology and Immunology, University of North Carolina School of Medicine, Chapel Hill, NC USA

**Keywords:** Anti-VEGF, Bevacizumab, Ranibizumab, Retinopathy of prematurity, Diabetic retinopathy, Age-related macular degeneration

## Abstract

Inhibition of vascular endothelial growth factor (VEGF) in retinopathy of prematurity (ROP) raises concerns for premature infants because VEGF is essential for retinovascular development as well as neuronal and glial health. This study tested the hypothesis that endothelial cell-specific knockdown of VEGF receptor 2 (VEGFR2), or downstream STAT3, would inhibit VEGF-induced retinopathy without delaying physiologic retinal vascular development. We developed an endothelial cell-specific lentiviral vector that delivered shRNAs to VEGFR2 or STAT3 and a green fluorescent protein reporter under control of the VE-cadherin promoter. The specificity and efficacy of the lentiviral vector-driven shRNAs were validated in vitro and in vivo. In the rat oxygen-induced retinopathy model highly representative of human ROP, the effects of endothelial cell knockdown of VEGFR2 or STAT3 were determined on intravitreal neovascularization (IVNV), physiologic retinal vascular development [assessed as area of peripheral avascular/total retina (AVA)], retinal structure, and retinal function. Targeted knockdown of VEGFR2 or STAT3 specifically in retinal endothelial cells by subretinal injection of lentiviral vectors into postnatal day 8 rat pup eyes efficiently inhibited IVNV, and knockdown of VEGFR2 also reduced AVA and increased retinal thickness without altering retinal function. Taken together, our results support specific knockdown of VEGFR2 in retinal endothelial cells as a novel therapeutic method to treat retinopathy.

**Electronic supplementary material:**

The online version of this article (10.1007/s10456-018-9618-5) contains supplementary material, which is available to authorized users.

## Introduction

Agents that inhibit the bioactivity of vascular endothelial growth factor (VEGF) have dramatically improved visual acuity outcomes in a number of otherwise blinding eye diseases, including diabetic retinopathy, age-related macular degeneration, neovascular glaucoma and retinal vein occlusions [1, 2]. The benefit of intravitreal delivery of either neutralizing antibodies to the ligand, VEGF, or fusion proteins that trap VEGF and other family members thereby preventing their binding and activation of receptors is best appreciated in adults, because the relatively small intravitreal dose is highly diluted in the large adult blood volume and reduces side effects associated with systemic intravenous anti-VEGF drug treatments [3–5]. However, in one condition, retinopathy of prematurity (ROP), an intravitreal injection of an anti-VEGF agent leads to high concentrations of the drug in the relatively small blood volume of the premature infant [6] and has raised concerns not only about inhibition of VEGF in the developing retina, but also about systemic inhibition of serum VEGF during organ system development in the premature infant [7]. Therefore, there is necessity in approaches that specifically inhibit VEGF where excessive signaling and pathology exist without reducing appropriate VEGF signaling in developing tissues or cells in the premature infant.

One approach to reduce the risk of systemic VEGF inhibition involves reducing the dose of neutralizing antibody. A de-escalating dose study of the anti-VEGF agent, bevacizumab, reported efficacy at inhibiting severe ROP in the short-term using 1/20th the dose originally reported in the Bevacizumab Eliminates the Angiogenic Threat (BEAT)-ROP study [8, 9]; however, serum VEGF was still reduced at this dose at 1 month. Other clinical studies assessed the role of another anti-VEGF agent, ranibizumab, which is more rapidly cleared than bevacizumab, in reducing severe ROP. In the Comparing Alternative Ranibizumab Dosages for Safety and Efficacy (CARE)-ROP study, ranibizumab was successful at reducing severe ROP at 24% the adult dose and did not reduce serum VEGF levels; however, some infants experienced recurrent ROP and required additional treatments of ranibizumab [10]. Recurrent severe ROP has been reported elsewhere with ranibizumab or bevacizumab treatment [11]. Also, it remains unknown what long-term outcomes and safety profiles will be. Therefore, better understanding of anti-VEGF effects is needed for safer treatments.

We approached the question of how to optimize anti-VEGF dose to safely inhibit retinopathy by specifically targeting upregulated VEGF in Müller cells in an experimental rat model of oxygen-induced retinopathy (OIR) that is highly representative of human ROP in that fluctuations in inspired oxygen cause a delay in physiologic retinal vascular development followed by intravitreal neovascularization (IVNV) and poor postnatal growth [12, 13], as seen in preterm infants with ROP. We developed cell-specific lentiviral gene therapy to knock down the VEGF ligand or a splice variant, VEGF_164_, to retinal levels of a room air raised pup of the same developmental age [14]. Despite efficacy at IVNV without adversely affecting physiological retinal vascular development, knockdown of Müller cell VEGF resulted in structural changes to the neural retina [15]. We also found that reduced VEGF secretion by Müller cells activated compensatory mechanisms for cell survival, which may have been a response from neural and glial cells that use VEGF as a survival mechanism in response to stress [16].

In this study, we developed and describe a novel method to knock down the angiogenic receptor of VEGF, VEGF receptor 2 (VEGFR2), specifically in retinal endothelial cells in the rat OIR model that represents human ROP [12]. Our goal was to regulate VEGF signaling rather than inhibit it, since VEGF signaling is necessary for retinal development, health and survival—knockout of a single VEGF allele or receptor is lethal [17–20]. We tested the hypothesis that specific knockdown of VEGFR2 in retinal endothelial cells would reduce retinopathy without interrupting ongoing physiologic retinal vascular development, reducing serum VEGF, interfering with postnatal growth or disrupting retinal structure or function. This is the first report to knock down gene expression specifically in retinal endothelial cells in rat to treat retinopathy.

## Materials and methods

### Generation of VE-cadherin lentiviral vector

**Fig. 1:**
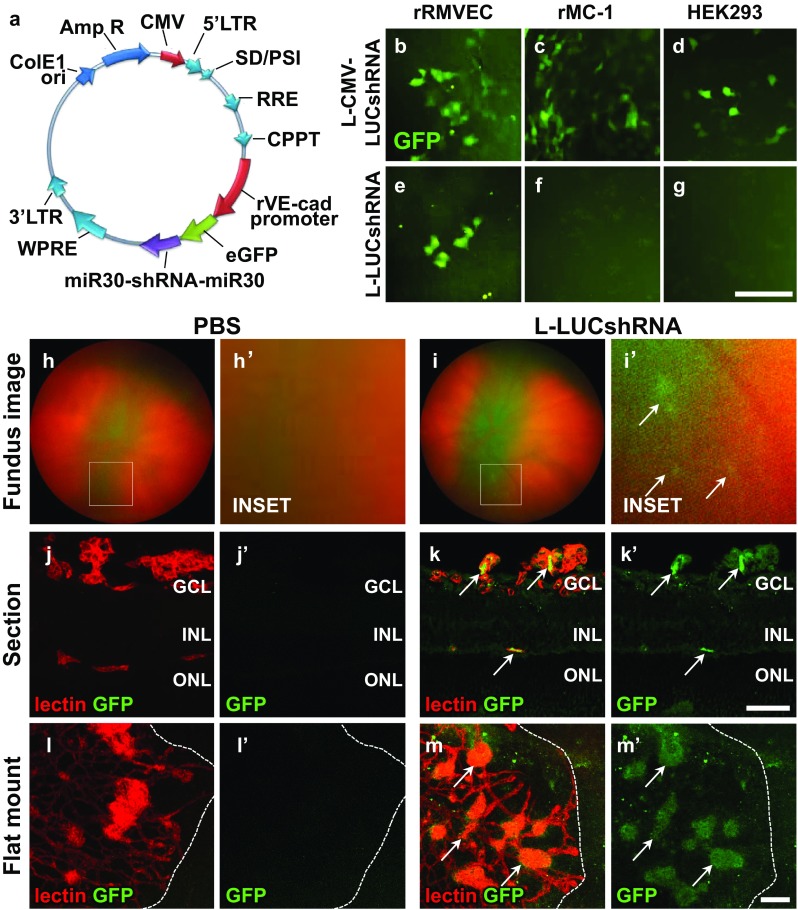
VE-cadherin lentiviral vector transduces rat endothelial cells in vitro and in vivo. **a** Diagram of plasmid containing the rat VE-cadherin promoter followed by GFP reporter and a microRNA30 embedded shRNA. **b–g** rRMVECs, rMC-1s, and HEK293 cells transduced with L-CMV-LUCshRNA or L-LUCshRNA. Transduction was visualized by GFP reporter. **h–i** Fundus images with Micron IV retina camera of OIR rat pups eyes treated with PBS or L-LUCshRNA. Insets are blown up in **h′** and **I′**. GFP expression was detected in L-LUCshRNA-treated retinas (arrows). **j–k** retinal sections and **l–m** flat mounts from OIR rats co-labeled with anti-GFP and lectin. GFP was localized to endothelial cells in L-LUCshRNA only (arrows). GFP channel is split out in **j′**–**m′**. Dotted lines in **l**–**m** delineate vascular versus avascular retina. Scale bar in *k’*= 50 µm; *m***′** = 100 µm. *Amp R* ampicillin resistance cassette, *CMV* cytomegalovirus; ColE1 ori, ColE1 origin of replication, *CPPT* central polypurine tract, *GCL* retinal ganglion cell layer, *GFP* green fluorescent protein, *HEK293* human embryonic kidney 293 cells, *INL* inner nuclear layer, *lectin* Isolectin-B4, *LTR* long terminal repeats, *miR30* microRNA30, *ONL* outer nuclear layer, *PBS* phosphate-buffered saline, *rMC-1* rat Müller cells, *RRE* Rev response element, *rRMVEC* rat retinal microvascular endothelial cells, *SD* splice donor, *shRNA* short hairpin RNA, *WPRE* woodchuck hepatitis virus (WHP) posttranscriptional regulatory element

We previously generated a lentiviral vector containing the CD44 promoter followed by a microRNA30 embedded shRNA and a GFP reporter [21]. In this study, the CD44 promoter was replaced by the VE-cadherin promoter (rat *Cdh5*) in a plasmid containing shRNA to luciferase and made into lentiviral vectors (L-LUCshRNA; Fig. 1a). We also generated a small amount of lentiviral vector using a plasmid in which the CD44 promoter was replaced by the CMV promoter (L-CMV-LUCshRNA). L-LUCshRNA and L-CMV-shRNA were used to initially test the endothelial cell specificity of the VE-cadherin promoter in vitro by transducing rRMVECs, rMC-1s, and HEK293 with lentiviral vector for 48 h at 2 × 10^6^ viral particles per ml. Lentiviral vectors for VEGFR2 (L-VEGFR2shRNA) and STAT3 (L-STAT3shRNA) were generated by replacing the luciferase shRNA sequence in the VE-cadherin plasmid with shRNA sequences to VEGFR2 or STAT3, respectively (Table 1). All plasmids were made into lentiviral vectors as previously described [21]. All methods were performed in accordance with the University of Utah Institutional Biosafety Committee for Biosafety Level II.

**Table 1:**
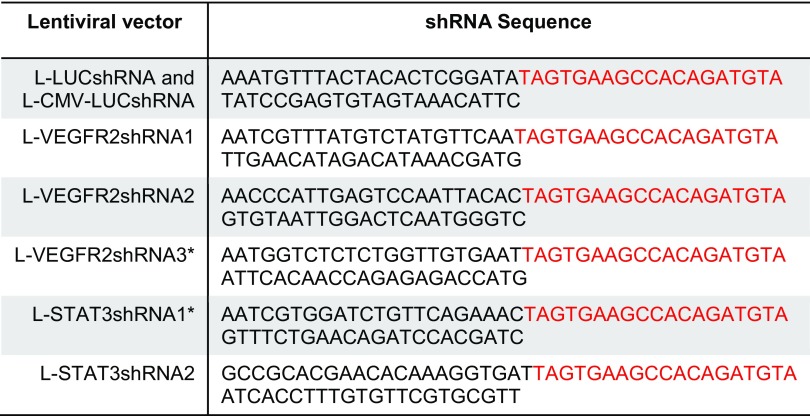
shRNA sequences inserted into plasmids

shRNA sequences were embedded into microRNA30 cassettes and made into lentiviral vectors. All vectors except L-CMV-LUCshRNA contained the VE-cadherin promoter

Black = shRNA sequence homology to the gene of interest. Red = loop sequence

*Denotes the vector that provided the greatest knockdown of the intended target as tested in rRMVECs and was used for experiments in this study

### Cell culture and viral transduction

rRMVECs (Cell Biologics, Chicago, IL) were maintained in EGM-MV (Lonza, Walkersville, MD) supplemented with 10% FBS, and cells between passage three and five were used for all experiments. To determine the knockdown efficiency of lenti-ve-cadherin delivered shRNA, rRMVECs were grown in 12-well dishes and transduced with L-LUCshRNA, L-VEGFR2shRNA, or L-STAT3shRNA at 2 × 10^7^ viral particles per ml. Media containing viral particles were removed after 12 h, and cells were maintained in 1% media to slow proliferation and prevent wells from becoming confluent. For real-time quantitative PCR (RT-qPCR) analysis, cells were directly harvested 72 h after transduction. For Western blot analysis, one transduced well from each group was trypsinized and passaged into a six-well plate. Passaged cells were grown for an additional 48 h, then starved in EBM medium overnight and treated with VEGF for 30 min for Western blots of phosphorylated VEGFR2 and STAT3, and for total VEGFR2 and STAT3.

### Real time-quantitative PCR (RT-qPCR) analysis

Seventy-two hours after transduction, cells were washed with PBS and then directly lysed and harvested using buffer RLT (Qiagen, Valencia, CA). mRNA was isolated using a Qiagen RNEASY kit, and cDNA was reverse transcribed from resulting samples using a High Capacity cDNA Reverse Transcription Kit (ThermoFisher Scientific, Waltham, MA). cDNA from each sample was analyzed using Taqman Gene Expression Assays (ThermoFisher Scientific) targeting *KDR* or *Stat3*, and the Δ*C*_T_ values for target genes were calculated using both *β-actin* and *TBP* as controls. ΔΔ*C*_T_ was calculated relative to the L-LUCshRNA control average, and figures were graphed using ${2^{ - \Delta \Delta {C_{\text{T}}}}}$ for each sample.

### Western blot analysis

Cells were lysed in RIPA buffer containing protease inhibitor cocktail (Roche Diagnostics, Indianapolis, IN) and orthovanadate (Thermo Scientifics, Rockford, IL), and clarified by centrifugation at 13,000×*g* rpm for 5 min at 4 °C. Protein concentration in the supernatant was quantified by bicinchoninic acid assay (BCA). 20 µg of protein from each treatment was loaded into NuPAGE 4–12% Bis-Tris Gels (Invitrogen, Carlsbad, CA), transferred to a PVDF membrane, and incubated with antibodies to phosphorylated VEGFR2 (p-VEGFR2, Y996) (1:500, Santa Cruz Biotechnology, Santa Cruz, CA), VEGFR2 (1:1000, Cell Signaling Technology, Danvers, MA), p-STAT3 (Y705) (1:1000, Cell Signaling Technology), or total STAT3 (1:1000, Cell Signaling Technology) at 4 °C overnight. After incubating with primary antibodies, membranes were then probed with HRP conjugated goat anti-rabbit secondary antibody or goat anti-mouse secondary antibody (1:3000–5000, ThermoFisher Scientific, Waltham, MA) at room temperature for 1 h. All the membranes were reprobed with HRP conjugated β-actin (1:3000, Santa Cruz Biotechnology) as loading controls. Densitometry analysis was performed on exposed films using UN-SCAN-IT software (Silk Scientific, Orem, UT) and normalized to β-actin.

### Rat OIR model

The rat OIR model of ROP was used as previously described [12]. Newborn Sprague Dawley rat pups, along with their nursing dam (Charles River Laboratories International, Wilmington, MA), were placed into an oxygen-controlled environment (Oxycycler, Biospherix) within 6 h of birth. Oxygen levels were cycled between 50 and 10% every 24 h for 14 days. At p8, as oxygen was changed from 10 to 50%, pups were administered bilateral subretinal injections of PBS, L-LUCshRNA, L-VEGFR2shRNA, or L-STAT3shRNA. At p14, rat pups were moved to room air. At p20, rat pups were weighed, sacrificed, and eyes were enucleated. All animal procedures were performed in accordance with protocols approved by the Animal Use and Care Committee at the University of Utah and in compliance with the Association for Research in Vision and Ophthalmology Statement for the Use of Animals in Ophthalmic and Visual Research. Treatments were randomized to the litters and each treatment was represented in a minimum of four different litters. Each litter contained control treatments and a mix of test treatments to allow comparisons across liters in the linear regression model. All rats used in this study received a unique identification number that was dissociated from those performing experiments until all of the data were collected in order to assure masking of investigators.

### Subretinal injections

At p8, rat pups received 1 µl bilateral subretinal injections of PBS, L-LUCshRNA, L-VEGFR2shRNA, or L-STAT3shRNA. Lentiviral vectors were administered at a dose of 1.0 × 10^9^ viral particles per ml. All injections contained sterile sodium fluorescein (1:10^6^, Akron Inc., Lake Forest, IL) to visualize injection location and to confirm that the solution entered the subretinal space with the dissecting microscope at the time of injection. Injections that were not in the subretinal space were noted and excluded from analyses.

### In vivo retinal imaging

Rats were anesthetized with intraperitoneal injections of ketamine and xylazine (75/10 mg kg^−1^ body weight). Pupils were dilated with tropicamide (1% solution; Bausch & Lomb Pharmaceuticals, Inc., Rochester, NY). Genteal gel (Novartis Pharmaceuticals Corp., East Hanover, NJ) was used as a contact agent and rat retinas were imaged with a fundus imaging camera (Micron IV, Phoenix Research Laboratories, Inc., Pleasanton, CA). Both bright field and GFP imaging were performed. Eyes with a damaged lens or detached retina due to subretinal injection were excluded from experiments. For eyes injected with lentiviral vector, only those expressing the GFP reporter confirmed by Micron IV imaging (Fig. 1i) were used for analyses of histology, flat mounts, or ERGs.

### Tissue handling

Enucleated rat eyes were fixed in 4% PFA for 30 min at room temperature (RT) with the cornea, lens, and iris removed or for 1 h RT with the cornea slit for most staining protocols. For tissue used for GFP staining, enucleated rat eyes were fixed in ice cold 4% PFA for 30 min and washed thoroughly in PBS, followed by removal of the cornea, lens, and iris before the eye cups were placed into HistoChoice Tissue Fixative (H2904, MilliporeSigma, Burlington, MA, USA) for 12–16 h at 4 °C. Retinas were dissected from fixed eyes and washed in PBS. At this point, retinas for flat mount were stained as described below. Retinas for sectioning were first cryopreserved by equilibrating them in a 30% sucrose solution and embedded in a 2:1 solution of optimal cutting temperature compound (OCT, Sakura Finetek USA, Inc., Torrance, CA, USA): 30% sucrose. Retinas were sectioned at 12 µm with a cryostat. Six sections were placed onto each charged microscope slide and each retina section was 120 µm from consecutive section.

### Immunostaining and Immunofluorescence

Retinal sections were first incubated in a 0.1% Triton Block (TB) solution (0.1% Triton x-100, 4% BSA, 5% NGS in PBS) for at least 30 min at 25 °C. Sections were then stained with mouse anti-GFP (75–132; 1:100; NeuroMab) primary antibody diluted in 0.1% TB for 8–12 h at 4 °C. Slides were then washed three times in PBS at 25 °C. Staining with goat anti-mouse (115-025-166; 1:100; Jackson ImmunoResearch) secondary antibody and lectin (Isolectin GS-IB4 from Griffonia simplicifolia, 1:200, I21411, Invitrogen, Grand Island, NY) diluted in 0.1% TB was performed at 25 °C for at least 3 h. Slides were washed again and mounted using Fluoromount-G (0100-20, SouthernBiotech, Birmingham, AL), which contains 4′,6-diamidino-2-phenylindole (DAPI). A similar procedure was performed for flat mounted retinas with the following modifications: a 0.4% TB solution was used; first incubation time was at least 12 h at 4 °C; primary and secondary incubations were 2–4 days at 4 °C; all washes were performed for at least 8 h (three PBS washes for a total of 8 h), and retinas were mounted using a DAPI free Fluoromount media (0100-01, SouthernBiotech).

### Microscopy

Microscope images were captured using an inverted Olympus Scanning laser confocal microscope (IX81, Olympus, Tokyo, Japan) or an inverted Olympus fluorescent microscope (IX81, Olympus). Any modifications to microscope images for publication were performed in either Adobe Photoshop (Adobe Systems Inc., San Jose, CA) or Microsoft Powerpoint (Microsoft Corporation, Redmond, WA) and were limited to image cropping and brightness/contrast changes that were uniformly done across the entire image.

### Flat mount analysis of AVA and IVNV

Retinas stained with lectin were flat mounted and imaged using an inverted fluorescence microscope. Whole retinal flat mount images were generated using Metamorph software (Molecular Devices, Inc., Sunnyvale, CA). Peripheral avascular/total retinal area (AVA) and intravitreal neovascular/total retinal area (IVNV) were measured by two independent and masked reviewers using FIJI software (National Institutes of Health, Bethesda, MD). For discrepancies, reviewers came to a consensus measurement after reexamination together prior to being unmasked to treatments. A minimum of 16 retinas were analyzed per treatment.

### Retinal thickness

Retinal sections were first permeabilized by incubating them in a 0.1% TB solution and stained by mounting with Fluoromount-G containing DAPI. Images for analysis were captured within one optical field from the optic nerve head using a scanning laser confocal microscope. Thickness of the retinal layers (GCL-IPL, INL, ONL, and Total) was measured using FIJI software. For each treatment, a minimum of 27 measurements were taken from at least 9 sections from three different rat eyes, except for L-STAT3shRNA treated eyes, in which only two rat eyes were used.

### Full-field Ganzfeld electroretinography (ERG)

Transduction of lentiviral vector was visualized at p20 with fundus imaging (Micron IV, PhoenixLabs); only transduced eyes were used for ERG experiments. Rats were dark-adapted overnight and handled under dim red light throughout the experiments. Rats were anesthetized with intraperitoneal injections of ketamine and xylazine (75/10 mg kg^−1^ body weight). Pupils were dilated with tropicamide (1% solution, Bausch & Lomb Pharmaceuticals), and Genteal gel (Novartis Pharmaceuticals Corp) was used as a contact agent. Rats were placed on a heating pad to maintain constant body temperature. Subdermal electrodes were placed at the base of the tail (ground electrode) and between the eyes (reference electrode). A corneal contact electrode was used to record ERGs. The LKC UTAS Visual Diagnostic System with BigShot Ganzfeld was used to record Ganzfeld ERG. The ERG was recorded using white light stimulus as previously described [15]. ERGs were recorded at p34. A minimum of nine eyes from different rats was analyzed.

### Serum VEGF ELISA

On p20, prior to euthanasia, blood samples were collected and placed at room temperature to allow blood to coagulate. After 2 h, clotted blood samples were spun down for 25 min at 2000×*g*, and the serum was collected. Serum samples were assayed as recommended using a rat VEGF ELISA from R&D systems (Minneapolis, MN).

### Statistical analysis

Based on previous studies [15], at least 16 flat mounts for AVA and IVNV are analyzed per group. A mixed effects linear regression model was used to statistically analyze data collected from OIR rats using STATA-14 software (StataCorp LLC, College Station, TX) that accounted for biological variation between litters and eyes. All data from OIR rats are presented as margins ± SEM that was generated by the linear regression model. For RT-qPCR, statistics were run using the non-transformed Δ*C*_T_ values. A Student’s *t* test was used to compare RT-qPCR and Western blot relative densitometry data. A *p* value ≤ 0.05 was considered statistically significant.

## Results

### Generation of vascular endothelial cell-specific lentiviral vector

We previously reported on the generation and use of a lentiviral vector containing a CD44 promoter that recruits polymerase II to drive expression of microRNA30 embedded short hairpin RNA (shRNA) and a GFP reporter specifically in Müller cells [21]. In order to specifically target retinal endothelial cells instead of Müller cells, we replaced the CD44 promoter with a rat endothelial cell-specific promoter, vascular endothelial cadherin (VE-cadherin, *Cdh5*), in a lentiviral vector containing shRNA to the non-mammalian gene luciferase (L-LUCshRNA), which serves as a control lentiviral vector (Fig. 1a). Both CD44 and VE-cadherin promoters recruit polymerase II to drive cell-specific expression of shRNAs that are embedded in a microRNA30 construct to increase transcription efficiency [22].

The ability of L-LUCshRNA to specifically target rat endothelial cells was initially tested in vitro by transducing rat retinal microvascular endothelial cells (rRMVECs), rat Müller cells (rMC-1s), and human embryonic kidney cells (HEK293) with either L-LUCshRNA or a lentiviral vector containing the constitutive cytomegalovirus (CMV) promoter to drive GFP expression (L-CMV-LUCshRNA). All cell types treated with L-CMV-LUCshRNA were transduced by the vector and expressed GFP (Fig. 1b–d), whereas only rRMVECs were transduced when treated with L-LUCshRNA (Fig. 1e–g). We further tested the specificity of L-LUCshRNA in vivo by administering subretinal injections of L-LUCshRNA and control PBS into postnatal day (p) 8 OIR rat pup eyes. Prior to euthanasia at p20, transduction was assessed by fundus imaging of the pup retinas using a Micron IV camera (Phoenix Research Laboratories, Inc.) and GFP filter set. GFP expression was visible in eyes that received subretinal injections of L-LUCshRNA, but not in those that received subretinal injections of PBS (Fig. 1h–i). In GFP and lectin co-labeled retinal cross sections and flat mounts from OIR rats injected with L-LUCshRNA, transduction was limited to endothelial cells in retinal vessels and intravitreal neovascularization (Fig. 1j–m). Together, these data show that our endothelial cell-specific lentiviral vector selectively transduced rat retinal endothelial cells both in vitro and in vivo.

### Lentiviral vector has no effect on retinal angiogenesis or retinal structure

**Fig. 2:**
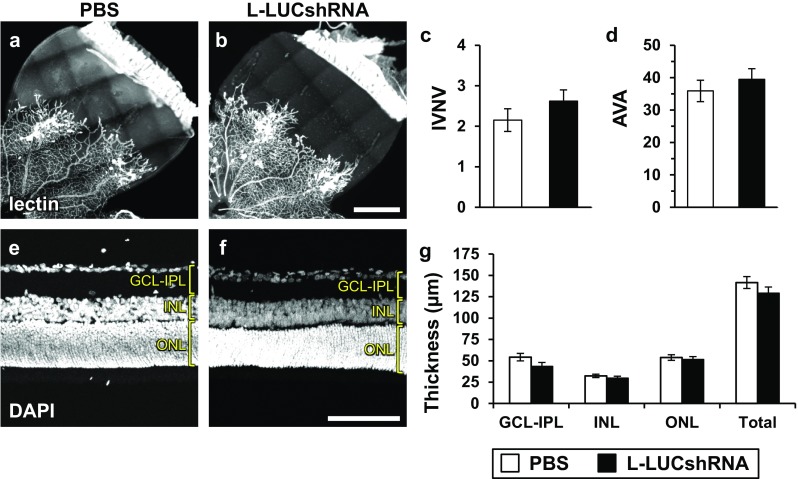
L-LUCshRNA has no effect on AVA, IVNV, or retinal thickness. **a, b** Representative flat mount images of PBS- or L-LUCshRNA-treated OIR eyes stained with lectin. **c** IVNV was not significantly different (PBS vs. L-LUCshRNA; 2.15 ± 0.28 vs. 2.62 ± 0.28%; *p* = 0.10; *n* = 32 or *n* = 36 retinas, respectively). **d** AVA was not significantly different (PBS vs. L-LUCshRNA; 35.90 ± 3.30 vs. 39.46 ± 3.29%; *p* = 0.15; *n* = 32 or *n* = 36 retinas, respectively). **e–f** Representative retinal cross section images of PBS- or L-LUCshRNA-treated OIR eyes stained with DAPI. **g** Retinal thicknesses were not significantly different (PBS vs. L-LUCshRNA; GCL-IPL: 54.28 ± 4.42 vs. 43.53 ± 4.51 µm, *p* = 0.10; INL: 32.29 ± 1.95 vs. 29.81 ± 2.05 µm, *p* = 0.38; ONL: 53.79 ± 3.28 vs. 51.57 ± 3.40 µm, *p* = 0.64; Total: 141.62 ± 6.99 vs. 129.19 ± 7.19 µm; *p* = 0.22; *n* = 33 or *n* = 27 measurements per layer, respectively). L-LUCshRNA images and data were also used as controls in Figs. 4 and 7. Scale bar in *b* = 500 µm; *f* = 100 µm. All data presented are adjusted margins ± SEM. *AVA* peripheral avascular/total retinal area, *DAPI* 4′,6-diamidino-2-phenylindole, *GCL* retinal ganglion cell layer, *INL* inner nuclear layer, *IPL* inner plexiform layer, *IVNV* intravitreal neovascular/total retinal area, *lectin* Isolectin-B4, *µm* micrometer, *ONL* outer nuclear layer, *PBS* phosphate-buffered saline

To test the effects of the endothelial cell-specific lentiviral vector on the rat OIR model, bilateral subretinal injections of L-LUCshRNA or PBS were administered to OIR pups at p8. At p20, eyes were screened for transduction with retinal imaging immediately before the eyes were harvested for analysis of IVNV and AVA or retinal structure in retinal sections. No significant differences were measured in IVNV (Fig. 2a–c, *p* = 0.10) or AVA (Fig. 2a–b, d, *p* = 0.15) between PBS- and L-LUCshRNA-treated eyes. There were also no significant differences measured in retinal layer or total retinal thicknesses between PBS- and L-LUCshRNA-treated eyes (Fig. 2e–g, *p* values: GCL-IPL = 0.10 INL = 0.38, ONL = 0.64, Total = 0.22). Taken together, these data demonstrate that the lentiviral vector has no effect on retinal angiogenesis or on the structure of the retinal layers in OIR rats at p20.

### Knockdown of VEGFR2 in retinal endothelial cells reduces pathologic IVNV, extends physiologic retinal vascular development, and increases retinal thickness

**Fig. 3:**
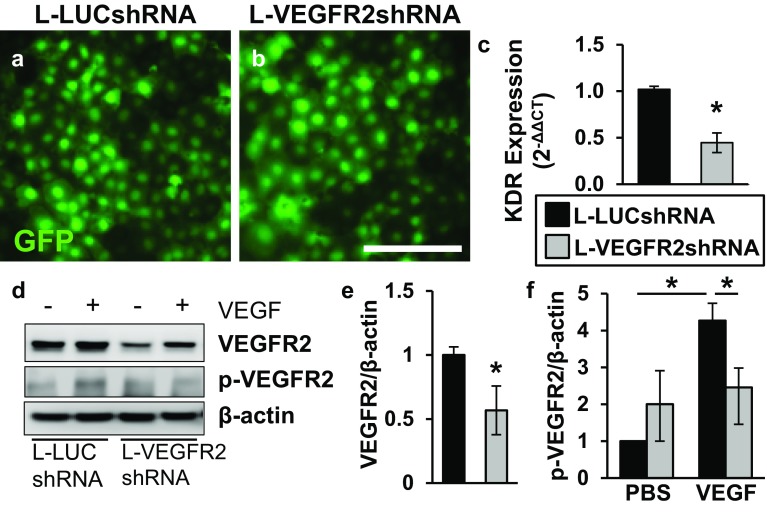
L-VEGFR2shRNA knocks down VEGFR2 mRNA and protein in rat endothelial cells. **a, b** rRMVECs transduced by L-LUCshRNA or L-VEGFR2shRNA for 72 h. **c** RT-qPCR, mRNA expression for VEGFR2 was reduced in rRMVECs transduced by L-VEGFR2shRNA, compared to L-LUCshRNA (L-LUCshRNA vs. L-VEGFR2shRNA; 1.02 ± 0.14 vs. 0.45 ± 0.11; *p* = 0.03; *n* = 2–4). **d** Image of Western blot gel from rRMVECs. **e, f** Densitometry quantification of total VEGFR2 protein (**e**) and p-VEGFR2 (**f**) relative to β-actin. **e** VEGFR2 protein was significantly reduced in rRMVECs transduced by L-VEGFR2shRNA, compared to L-LUCshRNA when cells were treated with PBS or VEGF (L-LUCshRNA vs. L-VEGFR2shRNA; *p* = 3.56 × 10^−4^; *n* = 6). **f** p-VEGFR2 protein was significantly increased in VEGF-treated rRMVECs transduced by L-LUCshRNA compared to PBS treatment (L-LUCshRNA + PBS vs. L-LUCshRNA + VEGF; *p* = 0.003; *n* = 3) that was reduced in rRMVECs transduced by L-VEGFR2shRNA (L-LUCshRNA + VEGF vs. L-VEGFR2shRNA + VEGF; *p* = 0.04; *n* = 3). Scale bar in *b* = 50 µm. Data are means ± SEM. *GFP* green fluorescent protein, *KDR* kinase insert domain receptor (VEGFR2 gene), *p-VEGFR2* phosphylated-VEGFR2

To evaluate the effect of endothelial cell-specific VEGFR2 knockdown in OIR, three different shRNA sequences targeting VEGFR2 (Table 1) were designed and inserted into the VE-cadherin plasmid and made into lentiviral vectors (L-VEGFR2shRNA). The L-VEGFR2shRNA providing the greatest VEGFR2 mRNA knockdown in rRMVECs was determined and used in subsequent experiments (data not shown). The efficiency of L-VEGFR2shRNA compared to L-LUCshRNA was tested in vitro by transducing rRMVECs with L-LUCshRNA or L-VEGFR2shRNA (Fig. 3a-b) and by assessing VEGFR2 mRNA and protein levels. The expression of VEGFR2 mRNA was significantly reduced in rRMVECs transduced by L-VEGFR2shRNA compared to L-LUCshRNA (Fig. 3c; 56% reduction, *p* = 0.03). Total VEGFR2 protein was significantly reduced in rRMVECs transduced by L-VEGFR2shRNA compared to L-LUCshRNA (Fig. 3e, *p* = 3.56 × 10^−4^). VEGF treatment significantly increased p-VEGFR2 protein in rRMVECs transduced by L-LUCshRNA compared to PBS-treated rRMVECs (Fig. 3f, *p* = 0.003), and this effect was reduced in rRMVECs transduced by L-VEGFR2shRNA (Fig. 3f, *p* = 0.04). Therefore, L-VEGFR2shRNA specifically transduced rat retinal endothelial cells and efficiently knocked down VEGFR2.

**Fig. 4:**
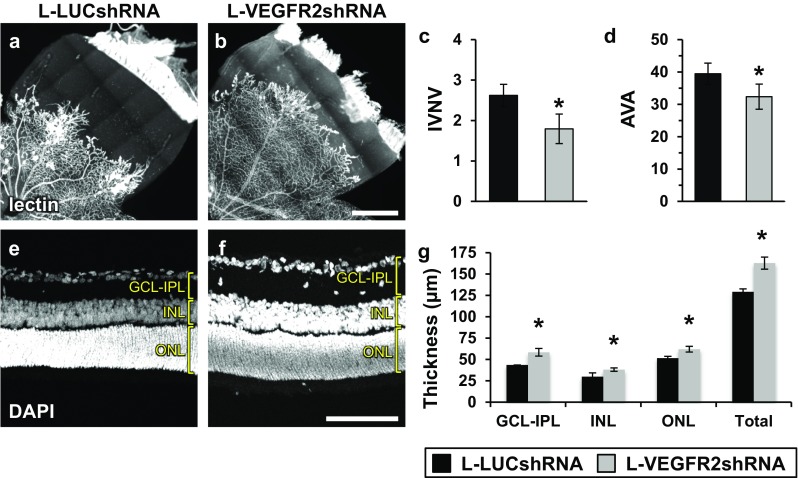
Knocked down VEGFR2 in retinal endothelial cells reduces IVNV and AVA and increases retinal thickness measurements. **a, b** Representative flat mount images of L-LUCshRNA- or L-VEGFR2shRNA-treated OIR eyes stained with lectin. **c** IVNV was significantly decreased in L-VEGFR2shRNA compared to control, L-LUCshRNA (L-LUCshRNA vs. L-VEGFR2shRNA; 2.62 ± 0.28 vs. 1.79 ± 0.36%; *p* = 0.03; *n* = 36 or 16 retinas, respectively). **d** AVA was significantly decreased in L-VEGFR2shRNA compared to control, L-LUCshRNA (L-LUCshRNA vs. L-VEGFR2shRNA; 39.46 ± 3.29 vs. 32.37 ± 3.89%; *p* = 0.03; *n* = 36 or *n* = 16 retinas, respectively). **e, f** Representative retinal cross section images of L-LUCshRNA- or L-VEGFR2shRNA-treated OIR eyes stained with DAPI. **g** Retinal thicknesses were significantly increased in L-VEGFR2shRNA compared to control, L-LUCshRNA for all measurements (L-LUCshRNA vs. L-VEGFR2shRNA; GCL-IPL: 43.53 ± 4.51 vs. 58.34 ± 4.45 µm, *p* = 0.02; INL: 29.81 ± 2.05 vs. 38.09 ± 2.00 µm, *p* = 0.004; ONL: 51.57 ± 3.40 vs. 61.96 ± 3.33 µm, *p* = 0.03; Total: 129.19 ± 7.19 vs. 162.82 ± 7.07 µm, *p* = 0.001; *n* = 27 or *n* = 30 measurements per layer, respectively). L-LUCshRNA images and data were also used as controls in Figs. 2 and 7. Scale bar in *b* = 500 µm; *f* = 100 µm. *Denotes significantly different from L-LUCshRNA (*p* < 0.05). All data presented are margins ± SEM. *AVA* peripheral avascular/total retinal area, *DAPI* 4′,6-diamidino-2-phenylindole, *GCL* retinal ganglion cell layer, *INL* inner nuclear layer, *IPL* inner plexiform layer, *IVNV* intravitreal neovascular/total retinal area, *lectin* Isolectin-B4, *µm* micrometer, *ONL* outer nuclear layer

To test our hypothesis that specific knockdown of VEGFR2 in retinal endothelial cells would reduce IVNV safely, we measured IVNV, AVA, and retinal thickness in p20 retinas of rat pups in the OIR model following p8 subretinal injections of either L-VEGFR2shRNA or L-LUCshRNA control. Compared to control L-LUCshRNA transduced retinas, there was a significant reduction in IVNV in retinas transduced by L-VEGFR2shRNA (Fig. 4a–c; 32% reduction; *p* = 0.03). There was also a reduction in AVA (Fig. 4a, b, d; 18% reduction; *p* = 0.03) in L-VEGFR2shRNA transduced retinas compared to control, L-LUCshRNA. In addition, significant increases in all of the retinal layers and total retinal thicknesses were detected in L-VEGFR2shRNA transduced retinas compared to L-LUCshRNA transduced retinas (Fig. 4e–g, *p* values: GCL-IPL = 0.02, INL = 0.004, ONL = 0.03, Total = 0.001). Taken together, these data demonstrate that reducing VEGFR2 specifically in retinal endothelial cells reduces pathologic angiogenesis into the vitreous (i.e., IVNV), extends physiologic retinal vascular development and results in an increase in thickness of retinal layers.

### Targeting VEGFR2 in retinal endothelial cells maintains retinal function and has no effect on serum VEGF concentration or pup growth

**Fig. 5:**
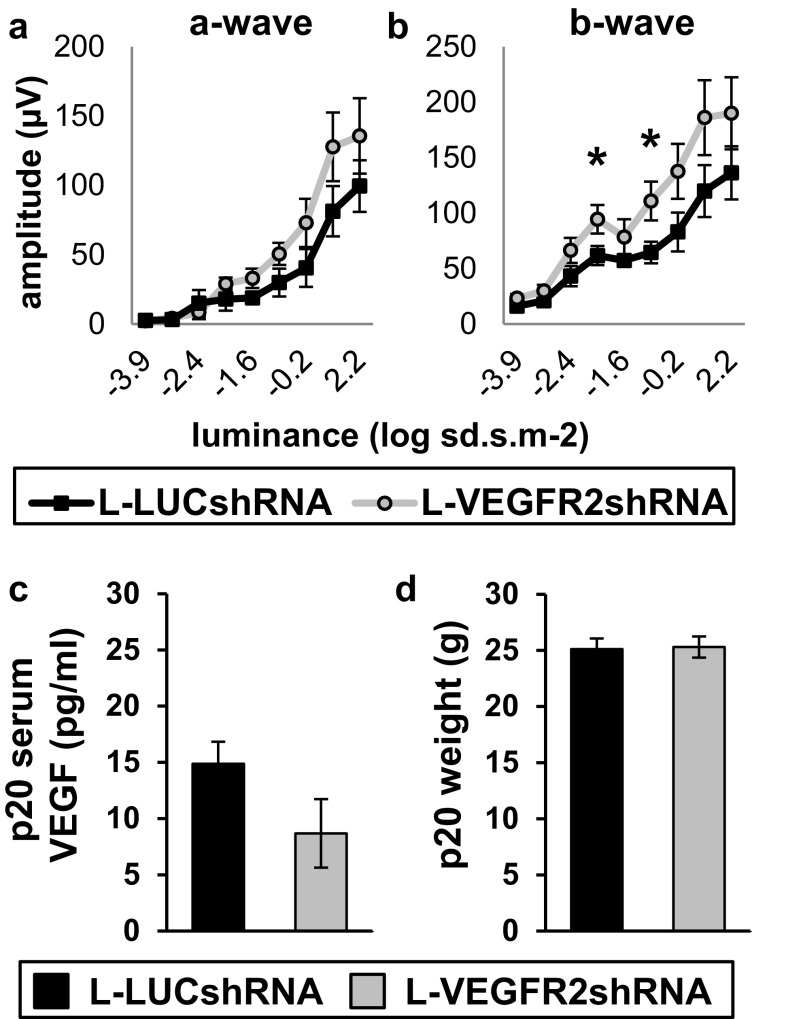
L-VEGFR2shRNA has no adverse effects on retinal function, serum VEGF, or pup weight. **a, b** Ganzfeld ERGs were used to record a- and b-wave amplitudes in OIR retinas transduced by L-LUCshRNA or L-VEGFR2shRNA. **a** Absolute a-wave amplitudes were not significantly different at any light stimulus intensity. **b** b-wave amplitudes were significantly increased at − 2 and − 1.2 log sd.s.m-2 light intensities in L-VEGFR2shRNA compared to control, L-LUCshRNA. **c** No significant difference in serum VEGF was measured in OIR pups (L-LUCshRNA vs. L-VEGFR2shRNA; 14.87 ± 1.97 vs. 8.69 ± 3.05 pg/ml; *p* = 0.08; *n* = 9 or *n* = 4, respectively). **d** p20 OIR pup weight was not significantly different in L-VEGFR2shRNA compared to control (L-LUCshRNA vs. L-VEGFR2shRNA; 25.12 ± 0.94 vs. 25.29 ± 1.04 g; *p* = 0.79; *n* = 26 or *n* = 12 pups, respectively). *Denotes significantly different from L-LUCshRNA (*p* < 0.05). All data presented are margins ± SEM. *g* grams, *ml* milliliter, *ONL* outer nuclear layer, *µV* microvolts

We next evaluated several measures of safety. The effects of endothelial cell VEGFR2 knockdown on retinal function were assessed by full-field Ganzfeld electroretinography (ERG) measured at p34. Both a- and b-wave amplitudes were maintained in the L-VEGFR2shRNA transduced retinas compared to L-LUCshRNA transduced retinas; some b-wave amplitudes increased at certain light stimuli (Fig. 5a, b, *p* values: − 2 log sd.s.m-2 = 0.05, − 1.2 log sd.s.m-2 = 0.04). Treatments with antibodies that sequester VEGF bioavailability can enter the blood stream and lower serum VEGF concentrations [6, 23, 24]. However, serum VEGF concentration at p20 in rats that received bilateral subretinal injections of L-VEGFR2shRNA was not statistically different from rats that received bilateral L-LUCshRNA (Fig. 5c, *p* = 0.08). Intravitreal anti-VEGF antibodies can also reduce postnatal growth of rat pups [25], but no differences in pup weight were detected at p20 between pups that received subretinal L-VEGFR2shRNA or L-LUCshRNA (Fig. 5d, *p* = 0.79). Taken together, these data demonstrate that regulation of VEGFR2 in retinal endothelial cells does not adversely affect retinal function, serum VEGF, or postnatal growth of rat pups.

### Knockdown of downstream STAT3 in retinal endothelial cells reduces pathologic IVNV and maintains retinal thickness, but has no effect on physiologic retinal vascular development

**Fig. 6:**
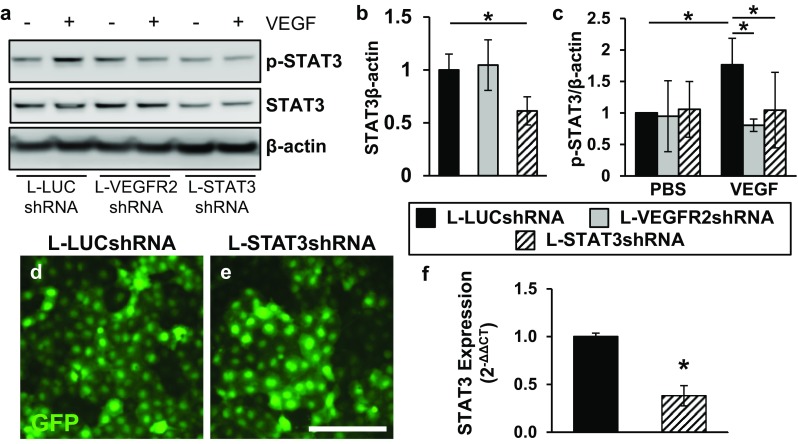
L-VEGFR2shRNA reduces VEGF phosphorylation of STAT3; L-STAT3shRNA knocks down STAT3 mRNA and protein in rat endothelial cells. **a** Image of Western blot from rRMVECs. **b** Total STAT3 protein was significantly reduced in rRMVECs transduced by L-STAT3shRNA compared to L-LUCshRNA when cells were treated with PBS or VEGF (L-LUCshRNA vs. L-STAT3shRNA; *p* = 3.08 × 10^−4^; *n* = 6). **c** p-STAT3 protein was significantly increased in VEGF-treated rRMVECs transduced by L-LUCshRNA compared to PBS treated (L-LUCshRNA + PBS vs. L-LUCshRNA + VEGF; *p* = 0.007; *n* = 3) that is reduced in rRMVECs transduced by L-VEGFR2shRNA or L-STAT3shRNA (L-LUCshRNA + VEGF vs. L-VEGFRshRNA + VEGF; *p* = 0.01; *n* = 3) (L-LUCshRNA + VEGF vs. L-STAT3shRNA; *p* = 0.04; *n* = 3). **d, e** rRMVECs transduced by L-LUCshRNA or L-STAT3shRNA. **f** RT-qPCR, mRNA expression for STAT3 was reduced in rRMVECs transduced by L-STAT3shRNA, compared to L-LUCshRNA (L-LUCshRNA vs. L-STAT3shRNA; 1.00 ± 0.03 vs. 0.38 ± 0.11; *p* < 0.001; *n* = 4–5). Scale bar in *e* = 50 µm. Data are means ± SEM. *GFP* green fluorescent protein, *p-STAT3* phosphylated-STAT3

The transcription factor, signal transducer and activator of transcription 3 (STAT3), is activated downstream of VEGF-induced activation of VEGFR2 and is important for VEGF-VEGFR2 induced rRMVEC proliferation [26]. In addition, pharmacologic inhibition of STAT3 significantly inhibits IVNV when VEGF is suppressed in a rat OIR model with supplemental oxygen [27]. These findings suggested that retinal endothelial cell STAT3 would be a reasonable target to safely inhibit IVNV without affecting the VEGF signaling pathways involved with physiologic retinal vascular development. We first addressed whether the L-VEGFR2shRNA significantly reduced STAT3 in rRMVECs. VEGF treatment of rRMVECs caused phosphorylation of VEGFR2 (p-VEGFR2) and STAT3 (p-STAT3), which was inhibited by transduction with L-VEGFR2shRNA but not control, L-LUCshRNA (Figs. 3f, 6a–c).

Next, two lentiviral vectors were created that delivered shRNA to STAT3 (L-STAT3shRNA), and the lentiviral vector providing the greater knockdown of STAT3 mRNA in rRMVECs was selected for subsequent experiments (Table 1 and data not shown). The knockdown efficiency of L-STAT3shRNA compared to L-LUCshRNA was then measured by mRNA expression or protein concentration in transduced rRMVECs (Fig. 6d, e). STAT3 mRNA was significantly reduced in rRMVECs transduced by L-STAT3shRNA compared to L-LUCshRNA (Fig. 6f, 62% reduction, *p* < 0.001). Total STAT3 protein was significantly reduced in rRMVECs transduced by L-STAT3shRNA compared to L-LUCshRNA (Fig. 6b, *p* = 3.08 × 10^−4^). VEGF treatment significantly increased p-STAT3 protein in rRMVECs transduced by L-LUCshRNA compared to PBS treated rRMVECs (Fig. 6c, *p* = 0.007), and this was reduced in rRMVECs transduced by L-STAT3shRNA (Fig. 6c, *p* = 0.04). We also measured *Stat3* or *KDR* mRNA in retinas transduced by L-LUCshRNA, L-VEGFR2shRNA, or L-STAT3shRNA. We recognize that endothelial cells make up a small proportion of cells in the retina, but nonetheless found significant reduction in *Stat3* specifically by L-STAT3shRNA and reduced *KDR* specifically by the L-VEGFR2shRNA (Suppl. Figure 1). Together, these studies confirmed that L-STAT3shRNA transduced rRMVECs and efficiently knocked down STAT3.

**Fig. 7:**
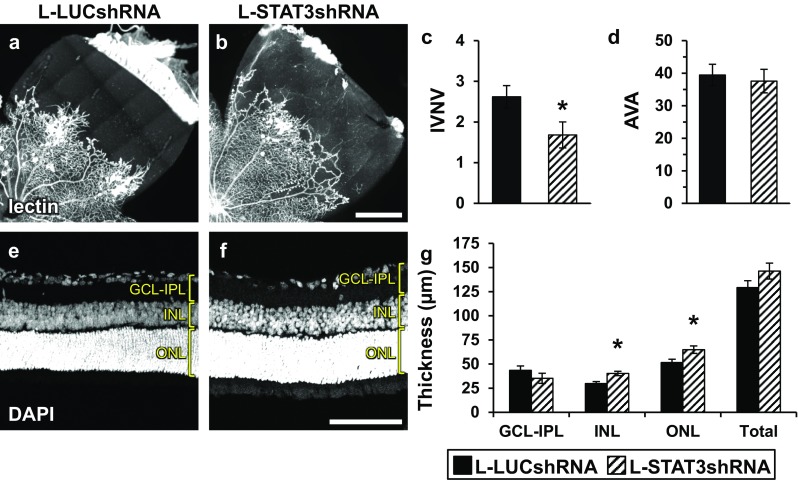
Knocked down STAT3 in retinal endothelial cells reduces IVNV, has no effect on AVA, and maintains retinal thickness. **a, b** Representative flat mount images of L-LUCshRNA- or L-STAT3shRNA-treated OIR eyes stained with lectin. **c** IVNV was significantly decreased in L-STAT3shRNA compared to control, L-LUCshRNA (L-LUCshRNA vs. L-STAT3shRNA; 2.62 ± 0.28 vs. 1.68 ± 0.32%; *p* = 0.003; *n* = 36 or *n* = 28 retinas, respectively). **d** AVA was not significantly different in L-STAT3shRNA compared to control, L-LUCshRNA (L-LUCshRNA vs. L-STAT3shRNA; 39.46 ± 3.29 vs. 37.57 ± 3.62%; *p* = 0.49; *n* = 36 or *n* = 28 retinas, respectively). **e, f** Representative retinal cross section images of L-LUCshRNA- or L-STAT3shRNA-treated OIR eyes stained with DAPI. **g** Retinal thickness measurements were significantly increased in L- STAT3shRNA compared to control, L-LUCshRNA for INL and ONL measurements only (L-LUCshRNA vs. L-STAT3shRNA; GCL-IPL: 43.53 ± 4.51 vs. 35.26 ± 5.26 µm, *p* = 0.23; INL: 29.81 ± 2.05 vs. 40.24 ± 2.25 µm, *p* = 0.001; ONL: 51.57 ± 3.40 vs. 64.90 ± 3.84 µm, *p* = 0.01; Total: 129.19 ± 7.19 vs. 146.29 ± 8.26 µm, *p* = 0.12; *n* = 27 or *n* = 27 measurements per layer, respectively). L-LUCshRNA images and data were also used as controls in Figs. 2 and 4. Scale bar in *b* = 500 µm; *f* = 100 µm. * denotes significantly different from L-LUCshRNA (*p* < 0.05). All data presented are margins ± SEM. *AVA* peripheral avascular/total retinal area, *DAPI* 4′,6-diamidino-2-phenylindole, *GCL* retinal ganglion cell layer, *INL* inner nuclear layer, *IPL* inner plexiform layer, *IVNV* intravitreal neovascular/total retinal area, *lectin* Isolectin-B4, *ONL* outer nuclear layer

To determine if selective knockdown of STAT3 in retinal endothelial cells affected IVNV and AVA, subretinal injections of L-STAT3shRNA or L-LUCshRNA were delivered bilaterally to p8 pup eyes. IVNV and AVA or retinal thickness was measured at p20 in successfully injected retinas. L-STAT3shRNA-treated retinas had a significant reduction in IVNV compared to L-LUCshRNA (Fig. 7a–c; 36% reduction; *p* = 0.003), but there was no significant difference in AVA (Fig. 7a, b, d, *p* = 0.49). Retinas transduced by L-STAT3shRNA had improved thickness of the INL and ONL but not in overall retinal thickness (Fig. 7e–g, *p* values: GCL-IPL = 0.23, INL = 0.001, ONL = 0.01, Total = 0.12). Taken together, these data suggest that activation of STAT3 in retinal endothelial cells by VEGFR2 is limited to pathologic IVNV and that extension of physiologic retinal vascular development by knockdown of endothelial VEGFR2 is through a separate mechanism.

## Discussion

In considering treatment for ROP, it is important to reduce IVNV and to protect or improve physiologic vascularity including physiologic retinal vascular development. A treatment that would increase physiologic retinal vascular development in the face of high oxygen and fluctuations in oxygenation and other stresses surrounding prematurity might prevent the later development of IVNV. It is also important to target treatment sufficiently in the fragile premature infants who undergo retinal and vascular development, as well as overall organ system development and maturation. We specifically knocked down endothelial cell VEGFR2 and safely reduced IVNV and extended physiologic retinal vascular development in an experimental model of ROP. Although it is not safe to perform subretinal injections in premature infants, these studies provide insights into mechanisms and safety in the management of ROP. Furthermore, the outcomes may translate to other retinopathies that are related to overactivation of VEGF signaling, including diabetic retinopathy.

As predicted based on our earlier studies [28–30], specific knockdown of retinal endothelial cell VEGFR2 with shRNA delivered by lentiviral vectors significantly reduced IVNV compared to control luciferase shRNA. Subretinal delivery of lentiviral vector affects about 1/3 of the retina and can be determined at the time of injection by the introduction of sodium fluorescein, which if in the vitreous cavity causes a much brighter fluorescence than if in the subretinal space. Only successfully injected eyes were used in analyses. Furthermore, we confirmed successful transduction of vessels by imaging GFP with the Micron IV retina camera. Although further confirmation of retinal endothelial cell-specific transduction was made by microscopic evidence of co-labeled anti-GFP and lectin, it was not possible to only measure IVNV in transduced areas of the retinal flat mounts. Despite this limitation, IVNV was significantly reduced by more than 30% in eyes treated with L-VEGFR2shRNA or L-STAT3shRNA, compared to L-LUCshRNA control. Since VEGF-VEGFR2 is important in physiologic and pathologic angiogenesis [17, 31], we predicted that the peripheral avascular retina might be unchanged or greater in the L-VEGFR2shRNA-treated eyes. However, physiologic retinal vascular development was extended in the L-VEGFR2shRNA-treated compared to L-LUCshRNA-treated eyes. We postulate several reasons. This may have been due to knockdown, but not knockout of VEGFR2, as evidenced by 56% reduction in VEGFR2 mRNA. We previously found that overactivation of VEGF signaling through VEGFR2 disordered developmental angiogenesis in an embryonic stem cell model, in which the gene, *flt1*, that encodes VEGFR1 was knocked out [32]. Inhibition of VEGFR2 ordered developmental angiogenesis in the rat OIR model by restoring the normal orientation of dividing endothelial cells [29, 30]. Previous investigators found that a VEGF trap to reduce binding to VEGFR1 or VEGFR2 caused persistent avascular retina in the beagle OIR model [33]. The VEGF trap bound VEGF and targeted VEGF signaling in all retinal and glial cells, not just the endothelial cells. (We expect VEGFR2 expression in neural and glial retina explains why the knockdown of retinal *KDR* by endothelial cell-specific L-VEGFR2shRNA was not significantly different from the L-LUCshRNA treated eyes.) Therefore, the evidence in our study suggests that knockdown of VEGFR2 specifically in retinal endothelial cells and not in all cells of the retina inhibits pathologic and permits extension of physiologic angiogenesis.

Knockdown of STAT3, which is downstream of VEGFR2, inhibited IVNV but did not affect physiologic retinal vascular development. We previously found that VEGF also activated STAT3 in Müller cells and that inhibition of STAT3 with an intravitreal inhibitor, AG490, reduced IVNV only when VEGF was reduced by supplemental oxygen [27]. We had interpreted this to be that inhibition of STAT3 was effective if targeted to endothelial cells (and not if Müller cell-STAT3 was affected), but another interpretation may be that reduced VEGF signaling through VEGFR2 extended physiologic retinal vascular development by a mechanism upstream of activated STAT3 or parallel to and independent from STAT3.

Both experimental lentiviral vectors thickened retinal layers compared to control, but a greater effect was seen with L-VEGFR2shRNA than with L-STAT3shRNA. This may be due to improved oxygenation of the retina through extension of physiologic retinal vascular development by L-VEGFR2shRNA or that VEGF normally bound by endothelial cell VEGFR2 was free to bind to neural and glial VEGF receptors that have been shown regulate cell survival and may be stressed by the OIR model [34–37].

In conclusion, we show that selective knockdown of VEGFR2 or STAT3 specifically in rat retinal endothelial cells using a novel gene therapy technique reduced IVNV. Knockdown of VEGFR2 in retinal endothelial cells also extended physiologic retinal vascular development. We believe this is the first report that specific targeting of retinal vascular endothelial cell mRNAs with shRNA has been done with gene therapy showing a biologic response. The ability to knock down and regulate signaling, rather than completely block it, has a potentially beneficial effect in translating to treatments for human diseases. We do not propose using subretinal drug delivery in human preterm infant eyes, but extensions of these techniques may be considered for adult proliferative retinal diseases. Future methods to target retinal endothelial cell VEGFR2, rather than the ligand VEGF, may benefit ROP, in which extension of physiologic retinal vascular development is desired. Additional studies are underway to understand molecular mechanisms from targeted VEGFR2 or STAT3 knockdown in retinal endothelial cells on neural and glial elements in the retina.

## Electronic supplementary material

Below is the link to the electronic supplementary material.

Supplementary material 1 (PDF 36 KB)
