# Correction to: Gene therapy knockdown of VEGFR2 in retinal endothelial cells to treat retinopathy

**DOI:** 10.1007/s10456-018-9626-5

**Published:** 2018-06-25

**Authors:** Aaron B. Simmons, Colin A. Bretz, Haibo Wang, Eric Kunz, Kassem Hajj, Carson Kennedy, Zhihong Yang, Thipparat Suwanmanee, Tal Kafri, M. Elizabeth Hartnett

**Affiliations:** 1 John A. Moran Eye Center, University of Utah, 65 N. Mario Capecchi Drive, Salt Lake City, UT 84132 USA; 2 Gene Therapy Center, University of North Carolina at Chapel Hill, Chapel Hill, NC USA; 3 Department of Microbiology and Immunology, University of North Carolina School of Medicine, Chapel Hill, NC USA

## Correction to: Angiogenesis 10.1007/s10456-018-9618-5

The article “Gene therapy knockdown of VEGFR2 in retinal endothelial cells to treat retinopathy”, written by “Aaron B. Simmons, Colin A. Bretz, Haibo Wang, Eric Kunz, Kassem Hajj, Carson Kennedy, Zhihong Yang, Thipparat Suwanmanee, Tal Kafri and M. Elizabeth Hartnett”, was originally published electronically on the publisher’s internet portal (currently SpringerLink) on 05 May 2018 without open access. With the author(s)’ decision to opt for Open Choice the copyright of the article changed on 20 June 2018 to © The Author(s) 2018 and the article is forthwith distributed under the terms of the Creative Commons Attribution 4.0 International License (http://creativecommons.org/licenses/by/4.0/), which permits use, duplication, adaptation, distribution and reproduction in any medium or format, as long as you give appropriate credit to the original author(s) and the source, provide a link to the Creative Commons license and indicate if changes were made. The original article has been corrected.

