# Supplementary material for: Gene therapy knockdown of VEGFR2 in retinal endothelial cells to treat retinopathy
Source: Angiogenesis. 2018 May 5;21(4):751–64. doi: 10.1007/s10456-018-9618-5 (PMC6203654; doi:10.1007/s10456-018-9618-5)
Supplement: Supplementary file 1 — Supplementary material 1 (PDF 36 KB) [file 10456_2018_9618_MOESM1_ESM.pdf]

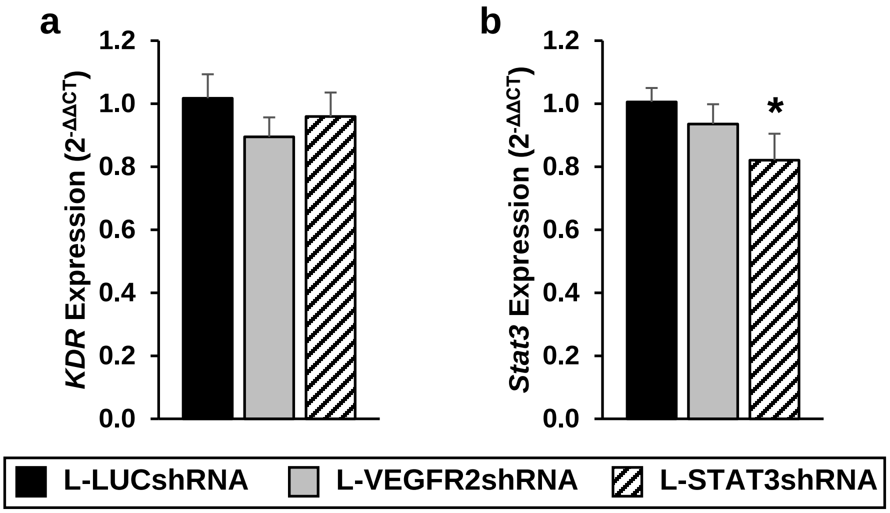


**Suppl. Fig. 1** Retinal mRNA expression data from retinas transduced by L-LUCshRNA, L-VEGFR2shRNA or L-STAT3shRNA. **a** Retinal *KDR* expression was reduced in L-VEGFR2shRNA transduced retinas compared to L-LUCshRNA (L-LUCshRNA vs. L-VEGFR2shRNA; ~11% reduction; p=0.204). **b** Retinal *Stat3* expression was significantly reduced in L-STAT3shRNA transduced retinas compared to L-LUCshRNA (L-LUCshRNA vs. L-STAT3shRNA; ~17% reduction; p=0.02).
